# Giant congenital lower lip nevus restored by local advanced skin flap: a case report

**DOI:** 10.1186/s13256-015-0515-x

**Published:** 2015-05-01

**Authors:** Liang Wang, Dongyun Yang, Liang Chen, Ling Tao, Jianyi Liu, Xia Dai, Shirong Li

**Affiliations:** Department of Plastic and Reconstructive Surgery, Southwest Hospital of Third Military Medical University, 30 Gaotanyan Main Street, Shapingba District, Chongqing, 400038 People’s Republic of China

**Keywords:** Nevus, Lip defect, Local advanced skin flap, Reconstruction

## Abstract

**Introduction:**

A congenital lower lip nevus is common, but a lower lip length lesion of more than 40% is hard to excise simply, without any distortion. We designed a lower lip bilateral advanced skin flap to restore the wound surface after completely and successfully removing the nevus.

**Case presentation:**

A 13-year-old Mongolian girl was referred to us with a giant congenital lower lip nevus. The lesion covered nearly half of her lower lip. We designed a lower lip bilateral advanced skin flap to restore the wound surface, after completely removing the nevus without advanced repair.

**Conclusions:**

This case report demonstrates a case of a lower lip length defect of more than 40%, repaired by direct closure without dysfunction. It was a successful attempt that minimized the subsequent scar and dysfunction.

## Introduction

Lower lip bilateral advanced skin flaps consist of sensate lip tissue with a minimized scar, but are generally restricted to the reconstruction of defects comprising of one third of the lower lip or less, in order to avoid microstomia. Reconstruction of larger defects usually involves Karapandzic flaps or other longer incisional advancement flaps, or free tissue transfer.

## Case presentation

**Figure 1:**
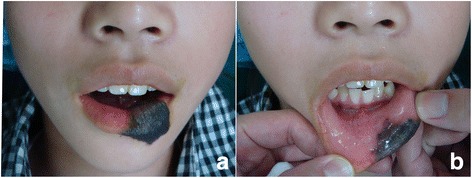
Preoperative view of lesion. **a**. the external surface. **b**. the inner surface.

In July 2011, a 13-year-old Mongolian girl was referred to us with giant congenital lower lip nevus. The lesion measured 3cm in the transverse diameter and covered nearly half of her lower lip. It had an even and hairy appearance, beginning at the median mouth mucosa of her left lower lip, extending inferiorly downward and encompassing much of her left lower white lip. The lesion was well circumscribed and asymptomatic (Figure 1). We removed it for esthetic purposes and its potential of forming malignancies.

**Figure 2:**
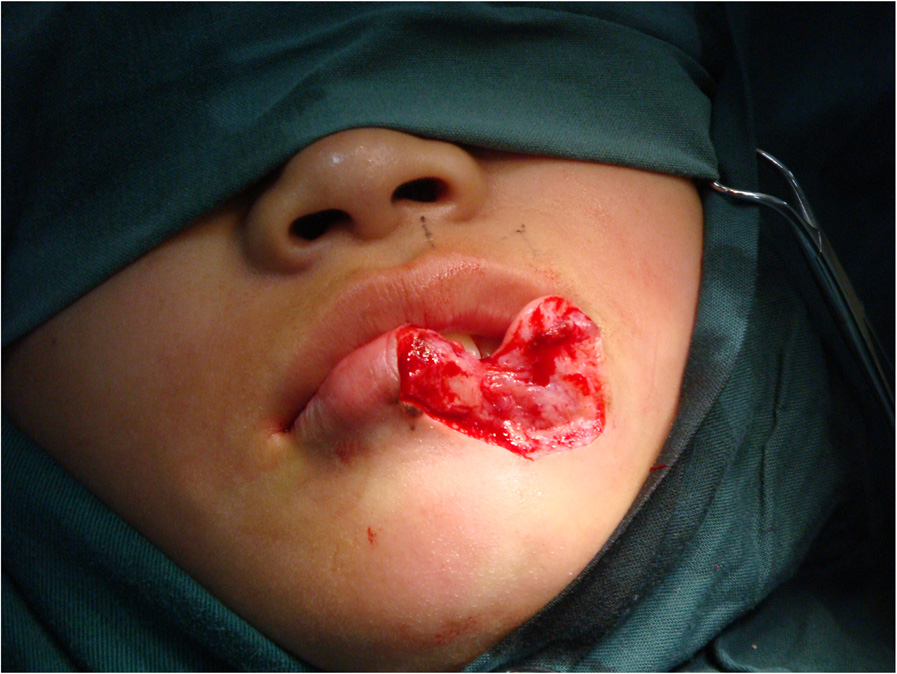
Intraoperative view of the lesion.

**Figure 3:**
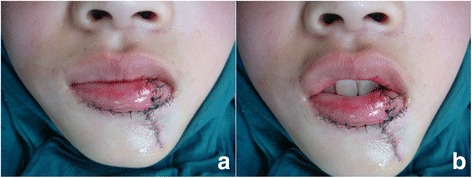
Immediately after the operation. **a**. the view of closed position of mouth. **b**. the view of opening position of mouth.

**Figure 4:**
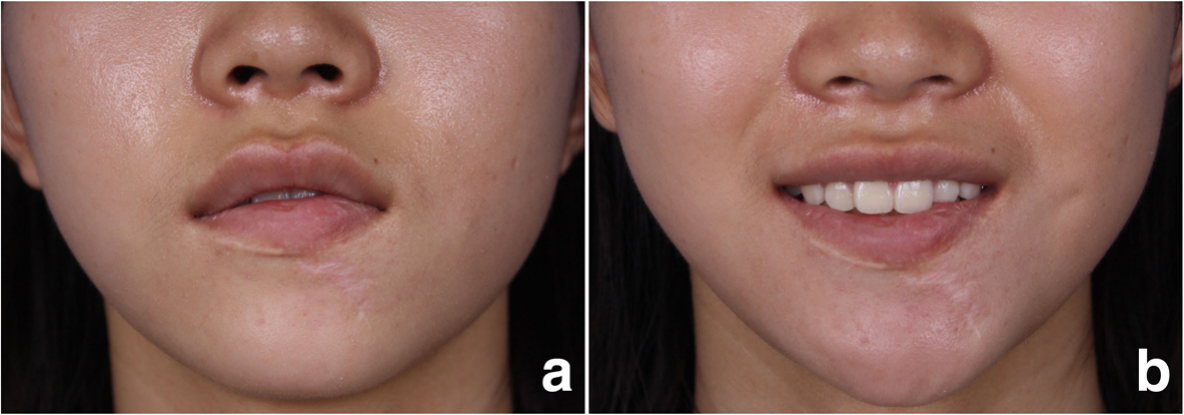
Results at 34 months postoperatively. **a**. the view of closed position of mouth. **b**. the view of smile.

We designed a lower lip bilateral advanced skin flap to restore the wound surface after completely removing the nevus; an Abbe flap was then prepared from her upper lip. Full-thickness wedge excision was performed on the lesion, including mucosa, orbicularis oris, and skin (Figure 2). We successfully used the bilateral advanced skin flap to restore the defect (Figure 3). The upper lip Abbe flap was not used. At 10 days postoperatively, her mouth opening was restricted, and the restriction was relieved 20 days later (at 30 days postoperatively). The results were satisfactory at 34 months postoperatively (Figure 4).

## Discussion

Since the mid-19th century, more than 200 different reconstruction techniques of the lower lip have been described [1]. The fact that so many techniques exist for lip reconstruction suggests the absence of one single method that is fully satisfactory and accepted by all [2]. The method with less postoperative distortion and scarring will be the superior method. In classic textbooks, direct closure of lip defects without distortion is possible, provided the defect involves one third or less of the lower lip length or one quarter of the upper lip [3,4]. If more than 30% of the lip tissue is missing, direct closure may not be possible and extensive constructive methods must be applied [5]. Only lip hemangiomas allow for a more aggressive resection, due to the significant displacement of the adjacent normal tissue, with less postoperative distortion than would traditionally be expected for nevi or malignancies [6].

Ebrahimi *et al*. [7] believed that in patients with a defect of 30 to 50% in size, and if the defect was located in central-lateral part of lower lip, a reversed Abbe flap from the upper lip and a step-ladder flap should be used. In patients with a defect of 50 to 80% in size, a bilateral Karapandzic flap and double reversed Abbe flap should be used. Roldán *et al*. [8] believed that a defect involving less than one third of the lip could be closed primarily after wedge excision. If the defect was up to two thirds of the lip in size, the Webster method, step technique, or such techniques combined with a cuneiform Abbe flap could be used for reconstruction of the lower lip. Doubtlessly, these extensive reconstructive methods will lead to more scarring in our patient. Therefore, the direct closure of a defect covering nearly half of the lower lip is a better choice, but a challenge for the surgeon.

With regards to the traditional treatment methods, we were unsure as to whether the defect could be restored by direct closure. Thus, we also designed the upper lip Abbe flap for advanced repair in case this would be necessary. The vermilion was easy to close after lesion excision was performed, while her white lip remained. An assisted incision was performed along the vermilion border of her lower lip, and the bilateral white-lipped skin flap was extensively undermined. In performing the repair in this manner, the defect was restored by a bilateral white-lipped advanced skin flap with fitness tension. After 30 days, her gape function was retained. In our case, we used the bilateral advanced skin flap to cover more than 40% of lost tissue on her lower lip successfully, without any dysfunction. This successful approach suggests a new way of performing major surgery of the lower lip.

## Conclusions

In conclusion, doctors may encounter such cases of lower lip length defects more than 40% in size, and because of traditional treatment methods, they tend to reconstruct the defect using the reversed Abbe flap taken from upper lip, the step-ladder flap, the Webster method, or other more invasive methods. We describe a case of a lower lip length defect of more than 40% in size, repaired by direct closure, without dysfunction. It was a successful attempt that minimized scarring and dysfunction. We hope that our case report will provide a new treatment option to consider when doctors are faced with such a tissue defect.

## Consent

Written informed consent was obtained from the patient and her mother for publication of this case report and any accompanying images. A copy of the written consent is available for review by the Editor-in-Chief of this journal.
